# Evaluation of the wound healing potential of isoquercetin-based cream on scald burn injury in rats

**DOI:** 10.1186/s41038-016-0032-1

**Published:** 2016-03-16

**Authors:** Nitish Bhatia, Gursharan Kaur, Varinder Soni, Juhi Kataria, Ravi K Dhawan

**Affiliations:** 1 Pharmacology Research Laboratory, Department of Pharmacology, Khalsa College of Pharmacy, Amritsar, India; 2 Department of Pharmaceutical Analysis, Khalsa College of Pharmacy, Amritsar, India; 3 Department of Biochemistry, Khalsa College of Pharmacy and Technology, Amritsar, India

**Keywords:** Isoquercetin, Scald, Burn, Free radicals, Wound healing

## Abstract

**Background:**

The present study was designed to evaluate the potential of isoquercetin-based cream formulation on scald burn wound injury in rats.

**Methods:**

Four isoquercetin-based cream formulations viz. 0.01, 0.02, 0.04, and 0.06 % *w*/*w* were prepared. Cream base and standard anti-burn cream containing silver sulfadiazine were also used for comparison. Scald burn was given to rats by pouring water at 90 °C on a shaved dorsal area of 20 mm^2^. Deep second-degree burn injury was produced which was evaluated for the next 21 days for the percentage of wound contraction and period of epithelialization. On day 21, the rats were sacrificed and histopathological slides were prepared using hematoxylin-eosin staining. Burned tissue was also screened for levels of oxidative stress using thiobarbituric acid reactive species (TBARS) and reduced glutathione (GSH) estimation.

**Results:**

There was a significant increase in the percentage of wound contraction and a significant decrease in the period of epithelialization in isoquercetin-based cream-treated groups as compared with the control group. However, most significant results were obtained with isoquercetin 0.06 % *w*/*w* cream. Histologically, isoquercetin 0.06 % *w*/*w* cream treatment resulted in almost complete re-epithelialization and re-structuring of the wound tissue. There was a significant rise in TBARS and a decrease in GSH levels in the burn injury group which was reversed to a major extent by the application of isoquercetin-based cream.

**Conclusions:**

The results indicate the wound healing potential of isoquercetin-based cream. Tissue biochemical studies indicate towards a possible role of free radical scavenging in the observed effects of isoquercetin in wound healing.

## Background

Burns can be defined as a detriment to the skin prompted by diverse sources, most frequently, extravagant heat or caustic chemicals. Burn injury may be distinguished as a kind of inflammation beneath the stratum corneum of the skin, which can either progress to extemporary healing or can deteriorate to further necrosis, contingent on the approach to treatment [1, 2].

Thermal burn injury tends to be the most common devastating trauma that emanates in either disability or death of burn patients [3]. Reactive oxygen species have been implicated in thermal burns that contribute to lipid peroxidation and other inflammatory response [4–6]. In contempt of the exploration of immense antiseptics, healing of burn wound still remains a challenge to modern medicine [7]. Current treatment strategies usually rely on topical application of the medicament with an aim to intensify wound healing, minimize inflammatory response, and most importantly halt the opportunistic infections that are commonly associated with severe wound injuries [8].

The most extensively used topical agent for burn injury includes silver sulfadiazine (SSD) 1 % cream, probably owing to its antimicrobial efficacy [9]. Regardless of this, the most significant clinical adverse effect of silver topical agent is the delayed wound healing along with other delineated adverse reactions such as resistance to silver sulfadiazine, renal toxicity, and leucopenia, therefore confirming that it should not be used on extensive wounds for extended periods [10, 11].

*Morus alba* L. (Mulberry) belonging to the Moraceae family, commonly known as white mulberry, has been reported to possess a wide number of bioactive flavanoids including quercetin, rutin, and isoquercetin. Isoquercetin being superior in concentration is the main anti-oxidant component of *M. alba* leaves that have been reported to manifest myriad of activities such as anti-inflammatory [12], anti-oxidant [13–15], antimicrobial [16], reducing lipid peroxidation both in vivo and in vitro [17], anti-diabetic activity [18], cytotoxic activity [19], and anti-hyperuricemic activity [20]. Despite the established anti-oxidant property of isoquercetin through diverse research [12–15], the potential of isoquercitrin in burn injury is not yet scrutinized. Therefore, the present study has been designed to evaluate the potential of isoquercetin-based cream preparations on scald burn injury in rats.

## Methods

### Animals

Wistar albino rats of either sex weighing 175 ± 25 g were employed in the present study. Animals were fed with standard laboratory feed (Kisan Feeds Ltd., Chandigarh, India) and water ad libitum. They were housed in the departmental animal house and were exposed to standard conditions of temperature (23 ± 2) °C, humidity of 50 ± 5 %, and natural cycles of light and dark. The experimental protocol was approved by the Institutional Animal Ethics Committee, and the care of the animals was carried out as per the guidelines of the Committee for the Purpose of Control and Supervision of Experimental Animals (CPCSEA), Ministry of Environment and Forests, Government of India (Reg. No.– 1753/PO/a/14/CPCSEA).

### Experimental protocol

The animals were divided into eight groups, each consisting of five rats. The duration of protocol was 21 days. The groups were assigned as follows:

- Group 1—normal/sham group
- Group 2—burn injury/control group
- Group 3—cream base-treated group
- Group 4—0.01 % soquercetin-based cream-treated group
- Group 5—0.02 % isoquercetin-based cream-treated group
- Group 6—0.04 % isoquercetin-based cream-treated group
- Group 7—0.06 % isoquercetin-based cream-treated group
- Group 8—silver sulfadiazine (standard) cream-treated group

### Experimental procedure

- Group 1—*normal/sham control group*: Animals in this group were just made to undergo the shaving procedure on the ear and then were kept undisturbed for the whole study protocol.
- Group 2—*burn injury/control group*: Animals were given scald burn injury. Animals were restrained in the rat holder, and 2 cm of the area on the back of the rats was carefully shaved to expose the skin. Hot water (90 °C) was poured over the shaved area for 10 s. This heat exposure caused a uniform second-degree burn on the skin. The animals were immediately resuscitated with an intra-peritoneal injection of ringer lactate solution (2 ml/100 g body weight) to prevent spinal shock.
- Group 3—*cream base-treated group*: Animals were given scalding burn injury as discussed in group 2. After the administration of ringer lactate solution, cream base (without any medicament) was applied on the affected area daily for a period of 21 days in an approximate time period between 10:00 and 11:00 AM with the help of a sterile gauge so as to cover the whole burned area uniformly.
- Group 4—*0.01 % isoquercetin-based cream-treated group*: The same procedure was applied as described in group 3, except that 0.01 % isoquercetin-based cream was used instead of cream base.
- Group 5—*0.02 % isoquercetin-based cream-treated group*: The same procedure was applied as described in group 3, except that 0.02 % isoquercetin-based cream was used instead of cream base.
- Group 6—*0.04 % isoquercetin-based cream-treated group*: The same procedure was applied as described in group 3, except that 0.04 % isoquercetin-based cream was used instead of cream base.
- Group 7—*0.06 % isoquercetin-based cream-treated group*: The same procedure was applied as described in group 3, except that 0.06 % isoquercetin-based cream was used instead of cream base.
- Group 8—*silver sulfadiazine (standard) cream-treated group*: The same procedure was applied as described in group 3, except that silver sulfadiazine cream (Silverine®) isoquercetin-based cream was used instead of cream base.

### Preparation and formulation of isoquercetin-based cream

Firstly, 0.4 g of liquid white paraffin, 1.5 g of stearyl alcohol, 0.6 g of solid white paraffin, propyl paraben (0.003 g), and isoquercetin (1, 2, 4, and 6 mg to formulate the desired concentrations of 0.01, 0.02, 0.04, and 0.06 %, respectively) were mixed and heated to a boiling point as aqueous phase. Meanwhile, 6.032 g of deionized water was added to the mixture of 1.4 g of propylene glycol, 0.06 g of sodium lauryl sulfate, and 0.005 g methyl paraben. The mixture was heated as organic phase. Then, two separate phases were mixed continuously while being treated to a constantly decreasing temperature. Thus, the uniform cream (10 g) was produced after cooling. The cream was filled within easily squeezable tube for application on the burned tissue of animals under experimental protocol. Our experimental research and formulations were carried out under sterile conditions. The final cream formulation was further tested for sterility on agar-based culture media to check the possibility of contamination with any pathogenic microbes.

### Pharmacological parameters

#### Epithelialization period

Epithelialization period was monitored by noting the number of days required for the eschar to fall off from the burn wound surface without leaving a raw wound behind.

#### Wound contraction

The size of lesions at 1, 11, and 21 days after burn injury was well apparent and determined by following the progressive changes in wound area planimetrically, excluding the day of the wounding. The burn wound surface area was then employed to calculate the percentage of wound contraction, taking the initial size of the wound 200 cm^2^ as 100 %, by using the following equation:

$$ \mathrm{Percentage}\kern0.5em \mathrm{of}\kern0.5em \mathrm{wound}\kern0.5em \mathrm{contraction}=\frac{\mathrm{Initial}\kern0.5em \mathrm{wound}\kern0.5em \mathrm{size}-\mathrm{Specific}\kern0.5em \mathrm{day}\kern0.5em \mathrm{wound}\kern0.5em \mathrm{size}}{\mathrm{Initial}\kern0.5em \mathrm{would}\kern0.5em \mathrm{size}}\times 100\% $$

#### Histopathology of the skin tissue

On day 21 after burn injury, the animals were sacrificed by decapitation after being anesthesized, burnt skin tissue samples were collected for histopathological examination. These tissue samples were fixed at 10 % neutral buffered formalin solution, embedded in paraffin wax, cut into 5-μm-thick sections, and stained with hematoxylin-eosin stain for examination by light microscopy. The slides were examined under an Olympus CX 41 microscope (Japan).

### Biochemical parameters

#### Estimation of thiobarbituric acid reactive species

The concentration of thiobarbituric acid reactive species (TBARS) in tissues was estimated by the method of Niehaus and Samuelson [21]. The tissue homogenate was prepared with Tris-HCl buffer, pH 7.5, and 0.1 ml of the supernatant was treated with 2 ml of (1:1:1 ratio) TBA-TCA-HCl reagent (0.37 % *w*/*v* thiobarbituric acid, 0.25 N HCl, and 15 % *w*/*v* TCA); the mixture was then kept in water bath for 15 min and further cooled and centrifuged for 10 min at room temperature. The absorbance was determined at 535 nm against the reference blank. The values were expressed as millimoles per 100 g of tissues.

#### Estimation of reduced glutathione

The concentration of glutathione (GSH) in tissues was estimated by the method of Ellman [22]. The supernatant (1 ml) was treated with 0.5 ml of Ellman’s reagent and 3.0 ml of phosphate buffer (0.2 M, pH 8.0). Ellman’s reagent was prepared by solubilizing 19.8 mg of 5, 5′-dithiobisnitro benzoic acid in 100 ml of 0.1 % sodium citrate. Finally, the absorbance was determined at 412 nm in a UV 1800 spectrophotometer (Shimadzu Corporation, Kyoto, Japan). Reduced GSH was expressed as milligrams per 100 g of tissues.

### Statistical analysis

The results were analyzed using one-way ANOVA followed by Tukey’s post hoc analysis with *p* ≤ 0.05 considered significant for all values.

## Results

### Tissue morphology

The burn wound tissue was characterized by pale white zone of tissue necrosis and associated inflammatory changes such as late edema and neutrophil infiltration.

### Wound contraction

A significant increase in the percentage of burn wound tissue contraction was prominent on day 1, day 11, and day 21 after burn injury (Table 1). The wound area was found to decrease significantly in isoquercetin (0.01, 0.02, 0.04, and 0.06 % *w*/*v*)-based cream-treated groups when compared to burn injury control group. Moreover, on day 11, the wound contraction was significantly greater in isoquercetin 0.06 % *w*/*v*-based cream group as compared to other groups for the same day (*p* < 0.05), which were comparable to the effect produced by silver sulfadiazine (standard drug)-treated group for the same day.

**Table 1:** Effect of cream base, silver sulfadiazine (standard), and isoquercetin-based creams on percentage of wound contraction in various animal groups under study after scald burn injury in the period of 21 days

Treatment groups	Percentage of wound contraction (mean ± standard deviations)		
	Day 1	Day 11	Day 21
Sham group	–	–	–
Burn injury group	0	3 ± 0.21	13 ± 0.46
Cream base-treated group	0	5 ± 0.24	14 ± 0.38
Silver sulfadiazine cream-treated group	0	25^a^ ± 1.23.	55^a^ ± 1.42
0.01 % isoquercetin cream-treated group	0	15^a^ ± 0.94	30^a^ ± 1.17
0.02 % isoquercetin cream-treated group	0	17^a^ ± 1.54	34^a^ ± 1.28
0.04 % isoquercetin cream-treated group	0	18^a^ ± 1.62	42^a^ ± 1.44
0.06 % isoquercetin cream-treated group	0	24^a^ ± 1.72	51^a^ ± 1.42

Results are represented as mean ± standard deviation

^a^Significant change in the mean value as compared to the burn injury group with *p* < 0.05

### Wound epithelialization

The mean period of epithelialization was found to decrease significantly in the isoquercetin (0.01, 0.02, 0.04, and 0.06 % *w*/*v*)-based cream-treated groups as compared to that in the burn injury group (Table 2). The decrease in the mean period of epithelialization in the isoquercetin 0.06 % *w*/*v*-based cream group was more significant as compared to that in the silver sulfadiazine (standard drug) group.

**Table 2:** Effect of different concentrations of isoquercetin-based creams on period of epithelialization after scald burn injury in a period of 21 days

Treatment groups	Period of epithelialization in days (mean ± standard deviations)
Sham group	–
Burn injury group	20.1 ± 0.54
Cream base-treated group	19.8 ± 0.48
Silver sulfadiazine cream-treated group	15.6 ± 0.44^a^
0.01 % isoquercetin cream-treated group	16.4 ± 0.46^a^
0.02 % isoquercetin cream-treated group	14.7 ± 0.42^a^
0.04 % isoquercetin cream-treated group	12.3 ± 0.36^a^
0.06 % isoquercetin cream-treated group	10.8 ± 0.27^a,b^

Results are represented as mean ± standard deviation

^a^Significant change in the mean value as compared to the burn injury group with *p* < 0.05

^b^Significant change in the mean value as compared to the silver sulfadiazine cream-treated group with *p* < 0.05

### Wound tissue histopathology

**Fig. 1:**
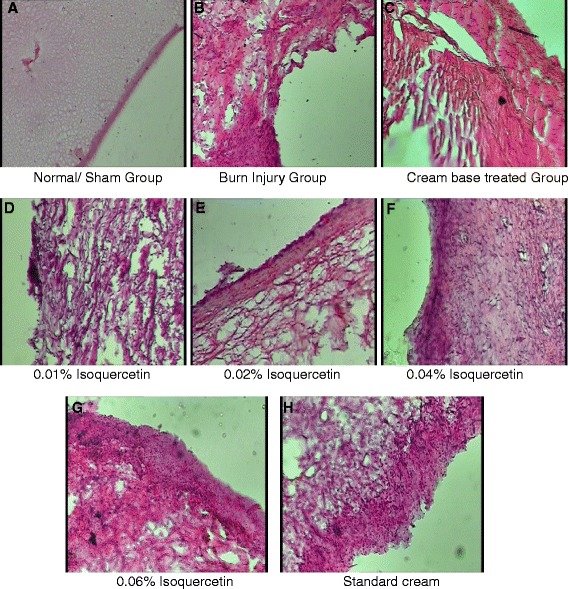
Representative histological slides of skin wound tissue of various groups on day 21 after burn injury. **a** Sham group with no histological changes; **b** burn injury group slide is characterized by incomplete formation of epithelial layer, inflammatory tissue damage, and edema; **c** cream base-treated group showing significant inflammatory damage and tissue edema; and **d** 0.01 % isoquercetin-based cream-treated group showing significant healing of burned tissue. The cellular structure of dermal layers has been restored. The signs of necrosis have been limited to most extent, and the tissue is devoid of any peri-vascular infiltration into tissue spaces. **e** 0.02 % isoquercetin-based cream-treated group showing more re-structuring and re-organizing of burned tissue. The process of tissue necrosis appears halted and tissue spaces less widened. **f** 0.04 % isoquercetin-based cream-treated group showing partial reformation of the epithelial layer with signs of tissue re-structuring and healing. **g** 0.06 % isoquercetin-based cream-treated group showing almost complete formation and healing of the epithelial layer. Moreover, there is less edematous swelling of tissue. **h** Standard (silver sulfadiazine) cream-treated group characterized by significant tissue repair and re-structuring with little edematous fluid

The skin tissue sections stained with hematoxylin and eosin stain were evaluated for histopathological changes that included modifications in the inflammatory cells and the magnitude of epidermal regeneration (Fig. 1). The granulation tissue sections of the isoquercetin-based cream-treated groups showed marked improvement in the wound healing in comparison with the control group. The sham group presented no pathological changes (Fig. 1a). The burn wound tissue sample exhibited massive necrotic areas and severe hemorrhage in dermis, and no epidermal regeneration was evident. Apart from this, a large number of infiltrated polymorphonuclear (PMN) cells were seen that were a clear indicator of severe inflammation (Fig. 1b). In the group treated with cream base, there was very less epidermal regeneration and PMN were also reduced with minute focal re-epithelialization and disorganized epidermal layers (Fig. 1c). In isoquercetin 0.01 and 0.02 % *w*/*v*-based cream-treated groups, there was relative regeneration of the epidermal layer along with fewer infiltrated PMN cells as compared to the control group (Fig. 1d, e). In isoquercetin 0.04 and 0.06 % *w*/*v*-based cream-treated groups, the epidermis was completely regenerated and well-organized with very slight PMN infiltration. The dermis layer was also not distorted with clear dermis papillae and clear demarcation between the epidermis and the dermis (Fig. 1f, g). In SSD (standard) cream-treated group, the epidermal regeneration was seen but with fewer epithelial cell layers and relatively disorganized. The PMN infiltration was also reduced, but some mast cells were seen in the dermis indicating mild inflammation (Fig. 1h).

### TBARS and GSH

**Fig. 2:**
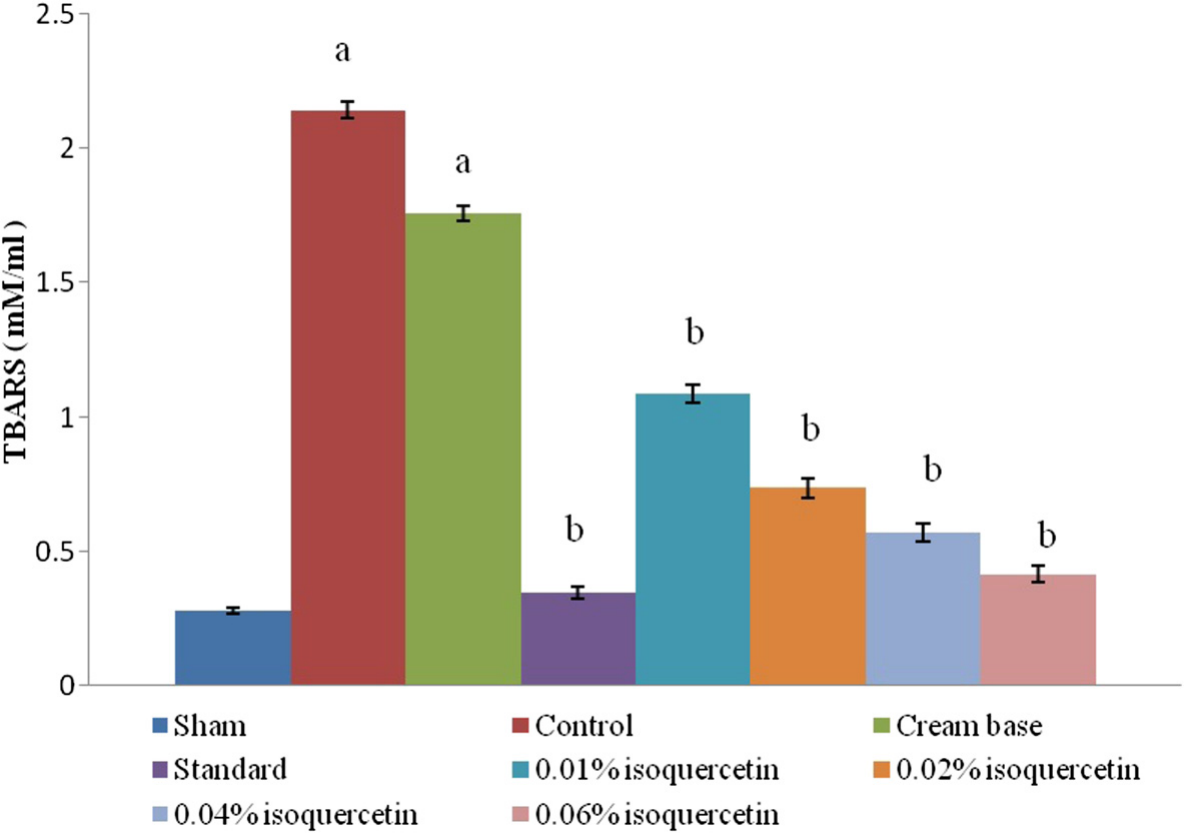
Changes in wound tissue TBARS after scald burn injury. The graph represents tissue levels of TBARS (mM/ml) in sham, burn injury control, cream base-treated, silver sulfadiazine (standard)-treated, and different isoquercetin-based cream-treated groups. Results are represented as the mean ± standard deviation; *n* = 6 per group. ^a^Significant change in the mean value as compared to the sham group with *p* < 0.05; ^b^significant change in the mean value as compared to the burn injury group with *p* < 0.05

**Fig. 3:**
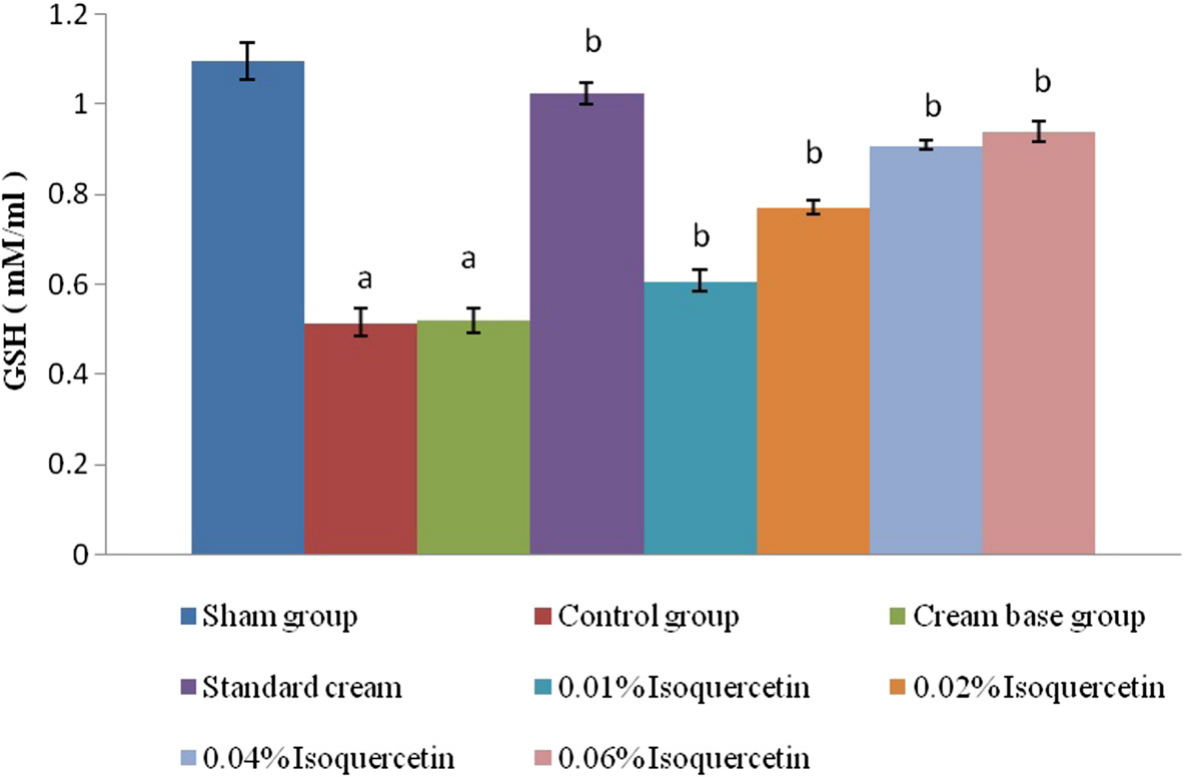
Changes in wound tissue GSH after scald burn injury. The graph represents tissue levels of GSH (mM/ml) of sham, burn injury control, cream base-treated, silver sulfadiazine (standard)-treated, and different isoquercetin-based cream-treated groups. Results are represented as the mean ± standard deviation; *n* = 6 per group. ^a^Significant change in the mean value as compared to the sham group with *p* < 0.05; ^b^significant change in the mean value as compared to the burn injury group with *p* < 0.05

The burn injury resulted in a significant increase in the wound tissue TBARS levels (Fig. 2) indicating a rise in oxidative stress, and a decrease in the level of GSH (Fig. 3) due to a compromising situation of a tissue anti-oxidant mechanism. The burn wound tissue when treated with isoquercetin-based cream depicted that there was a significant decrease in the tissue TBARS levels and a significant increase in GSH levels as compared to the control group. However, the isoquercetin 0.04 and 0.06 % *w*/*v*-based cream produced a more striking and significant response.

## Discussion

The anomaly of free radical arbitrated cell injury is customary to the burn injury. Burn injury has been proclaimed in the medical literature to provoke the formation and release of oxidative free radicals and other pro-inflammatory mediators that mainly contribute to lipid peroxidation [4–6]. The elevated lipid peroxidation level has been evident from the thiobarbituric acid reaction [23–27]. Thus, it may be suggested that the restoration of circulating volume by aggressive fluid resuscitation as well as the compensation of the loss of endogenous scavenging mechanisms may collectively produce significant oxidative stress. Many plants and their derivatives have demonstrated their proficiency as anti-oxidants in scavenging as well as curbing the release of the reactive oxygen species from the ethno-pharmacological perspective [28–31]. The *M. alba* leaf extract has been investigated for its anti-oxidant property using various methods like the iron (III)-reducing capacity, the total anti-oxidant capacity, the DPPH (2,2-diphenyl-1-picrylhydrazyl) radical scavenging activity, and an in vitro inhibition of ferrous sulfate-induced oxidation of lipids [32]. Isoquercetin being the main component of *M. alba* leaf was evaluated for its potential in healing the scald burns.

The burn wound recovery was assessed in terms of percentage of wound contraction and development of epithelial layer (period of epithelialization) after the eschar fell from the wound. A significant increase in the percentage of wound contraction in isoquercetin cream-treated group indicates the potent healing effect exerted by isoquercetin as compared to the control group. Furthermore, the dermal recovery of the tissue was also demonstrated in the wound as there was a highly significant decrease in the period of epithelialization in isoquercetin cream-treated group. Furthermore, it was interesting to observe that the decrease in the period of epithelialization in isoquercetin cream-treated group was more marked than that of silver sulfadiazine (standard)-treated group.

Moreover, the isoquercetin cream-treated groups exhibited a prominent decrease in the burn wound tissue TBARS levels and a significant rise in GSH levels in comparison with the burn injury group. An increase in the tissue TBARS levels is a clear indicator of an increase in the number of reactive oxygen species (ROS) in the burn wound tissue. Furthermore, a decrease in the GSH levels in the burn wound tissue points towards a progressive decrease in the anti-oxidant capacity of the skin tissue under the influence of burn injury.

The histopathological study of the burn wound tissue clearly demonstrated the improvement in the tissue necrosis and associated inflammatory changes in different concentrations of isoquercetin-based cream-treated groups. However, significant injury repair and regeneration of epithelium was evident in the 0.06 % *w*/*v* isoquercetin-based cream-treated group as compared to the burn injury group and other groups as well indicating that higher concentrations of isoquercetin are capable of reversing the burn injury-induced morphological and biochemical as well as micro-vascular changes implicated in necrosis.

The results indicate the potential of isoquercetin in halting and to a much extent reversing the pathological interplay of inflammatory mediators and associated pro-oxidative pathways at the site of burn injury. The observed actions could possibly be attributed to potent anti-oxidant effect showed by isoquercetin as evident by a significant decrease in the burn tissue TBARS levels in the isoquercetin cream-treated groups. However, as burn injury is always considered an acute dermatological insult representing a plethora of oxidative changes within a short span of time, therefore, isoquercetin-based formulation could be of immediate use in burn injury so as to suppress free radical production and oxidative stress as early as possible. As a phytoconstituent, isoquercetin is found in many plant species in different concentrations which indicates the phytochemical importance of this constituent in medicinal plants of higher order. We are of the opinion that in future isoquercetin could be favorably utilized by the pharmaceutical and cosmetic industry for the development of anti-burn injury formulations for possible use as supportive therapy to ongoing and prevalent burn management therapeutic regime.

## Conclusions

We can conclude that in a burn wound model in rats, isoquercetin-based cream was found to shorten the healing process both histopathologically and statistically as compared to SSD and the control group. Through its evident anti-oxidant and anti-inflammatory effects, isoquercetin can be used as an adjunctive or alternative agent to existing wound healing therapies in the future. However, wound tissue healing is a multi-factorial process involving a plethora of enzymes, receptors, and sub-molecular events. Therefore, further research is needed for highlighting the role of these processes and on the molecular mechanism of observed action of isoquercetin.

## References

[1] Monafo W, Bessy P, Herndon D (2001). Wound care in total burn care. Total burn care.

[2] Williams W, Herndon D (2001). Pathophysiology of the burn wound. Total burn care.

[3] McGill V, Kowal-Vern A, Fisher SG, Kahn S, Gamelli RL (1995). The impact of substance use on mortality and morbidity from thermal injury. J Trauma.

[4] Piccolo MT, Wang Y, Till GO (1999). Chemotactic mediator requirements in lung injury following skin burns in rats. Exp Mol Pathol.

[5] Singh V, Devgan L, Bhat S, Milner SM (2007). The pathogenesis of burn wound conversion. Ann PlastSurg.

[6] Rani M, Martin G, Schwacha MG (2012). Aging and the pathogenic response to burn. Aging and Disease.

[7] Gore MA, Akholekar D (2003). Evaluation of banana leaf dressing for partial-thickness burn wounds. Burns.

[8] Heggers J, Hawkins H, Edgar P, Villarreal C, Herndon D, Herndon D (2001). Treatment of infections in burns. Total burn care.

[9] Vloemans AFPM, SoesmanAM SM, Kreis RW, Middelkoop E (2003). A randomized clinical trial comparing a hydrocolloid-derived dressing and glycerol preserved allograft skin in the management of partial thickness burn. Burns.

[10] Klasen HJ (2000). A historical review of the use of silver in the treatment of burns. II. Renewed interest for silver. Burns.

[11] Atiyeh BS, Costagliola M, Hayek SN, Dibo S (2007). Effect of silver on burn wound infection control and healing: review of the literature. Burns.

[12] Rogerio AP, Kanashiro A, Fontanari C, Silva EV, Lucisano-Valim YM, Soares EG (2007). Anti-inflammatory activity of quercetin and isoquercitrin in experimental murine allergic asthma. Inflamm Res.

[13] Cornard JP, Boudet AC, Merlin JC (1999). Theoretical investigation of the molecular structure of the isoquercitrin molecule. J MolStruct.

[14] Lee S, Park HS, Notsu Y, Ban HS, Kim YP, Ishihara K (2008). Effects of hyperin, isoquercitrin and quercetin on lipopolysaccharide induced nitrite production in rat peritoneal macrophages. Phytother Res.

[15] Jung SH, Kim BJ, Lee EH, Osborne NN (2010). Isoquercitrin is the most effective antioxidant in the plant Thujaorientalis and able to counteract oxidative induced damage to a transformed cell line (RGC-5 cells). NeurochemInt.

[16] Razavi SM, Zahri S, Zarrini G, Nazemiyeh H, Mohammadi S (2009). Biological activity of quercetin-3-O-glucoside, a known plant flavonoid. BioorgKhim.

[17] Li R, Yuan C, Dong C, Shuang S, Choi MM (2011). In vivo antioxidative effect of isoquercitrin on cadmium-induced oxidative damage to mouse liver and kidney. NaunynSchmiedebergs Arch Pharmacol.

[18] Zhang R, Yao Y, Wang Y, Ren G (2011). Antidiabetic activity of isoquercetin in diabetic KK –Ay Mice. Nutrition & Metabolism.

[19] Vellosa JCR, Regasini LO, Khalil NM, Bolzani VS, Khalil OAK, Manente FA (2011). Antioxidant and cytotoxic studies for kaempferol, quercetin and isoquercitrin. EclQuím São Paulo.

[20] Hunyadi A, Liktor-Busa E, Márki A, Martins A, Jedlinszki N, Hsieh TJ, Báthori M, Hohmann J, Zupkó I. Metabolic effects of mulberry leaves: exploring potential benefits in type 2 diabetes and hyperuricemia. Evid Based Complement Alternat Med. 2013;2013:948627. doi: 10.1155/2013/948627. Epub 2013 Dec 5. 10.1155/2013/948627 PMC3870074 24381639

[21] Niehaus WG, Samuelson B (1968). Formation of MDA from phospholipid arachidonate during microsomal lipid peroxidation. Eur J Biochem.

[22] Ellman GL (1959). Tissue sulfhydryl groups. Arch BiochemBiophys.

[23] Fantone JC, Johnson KJ, Till GO, Ward PA (1983). Acute and progressive lung injury secondary to toxic oxygen products from leukocytes. Chest.

[24] Ward PA, Till GO, Hatherill JR, Annesley TM, Kukel RG (1985). Systemic complement activation, lung injury, and products of lipid peroxidation. J Clin Invest.

[25] Demling RH, La Londe C. Systemic lipid peroxidation and inflammation induced by thermal injury persists into the post-resuscitation period. J Trauma. 1990a;30:69–74. 10.1097/00005373-199001000-00010 2296069

[26] Demling RH, La Londe C. Early post burn lipid peroxidation: effect of ibuprofen and allopurinol. Surgery. 1990b;1007:85–93. 2296760

[27] Murray JD, Nelms CD, Kaufman TM, Horton JW (1992). Lipid peroxidation in plasma and heart tissue after burn injury. FASEB J.

[28] Haycock JW, Ralston DR, Morris B (1997). Oxidative damage to protein and alterations to antioxidant levels in human cutaneous thermal injury. Burns.

[29] Horton JW (2003). Free radicals and lipid peroxidation mediated injury in burn trauma: the role of antioxidant therapy. Toxicology.

[30] Ivona NC, Novac M, Maria V (2011). Risk factors influencing the oxidative stress in surgical therapy of skin grafts. Current Health Sciences Journal.

[31] Shinde A, Ganu J, Naik P (2012). Effect of free radicals & antioxidants on oxidative stress: a review. Journal of Dental & Allied Sciences.

[32] Arabshahi-Delouee S, Urooj A (2007). Antioxidant properties of various solvent of mulberry (Morusindica L.) leaves. Food Chem.

